# Genome-wide association study reveals quantitative trait loci for waterlogging-triggered adventitious roots and aerenchyma formation in common wheat

**DOI:** 10.3389/fpls.2022.1066752

**Published:** 2022-11-23

**Authors:** Le Xu, Chenchen Zhao, Jiayin Pang, Yanan Niu, Huaqiong Liu, Wenying Zhang, Meixue Zhou

**Affiliations:** ^1^ MARA Key Laboratory of Sustainable Crop Production in the Middle Reaches of the Yangtze River (Co-construction by Ministry and Province), College of Agriculture, Engineering Research Centre of Ecology and Agricultural Use of Wetland, Ministry of Education, Yangtze University, Jingzhou, China; ^2^ Tasmanian Institute of Agriculture, University of Tasmania, Launceston, TAS, Australia; ^3^ The UWA Institute of Agriculture and School of Agriculture and Environment, The University of Western Australia, Perth, WA, Australia

**Keywords:** waterlogging, wheat, aerenchyma, ethylene, adventitious roots

## Abstract

Waterlogging severely affects wheat growth and development. Limited availability of oxygen in the root zone negatively affects the metabolism of plants. The formation of adventitious roots (ARs) and root cortical aerenchyma (RCA) are the most important adaptive trait contributing to plants’ ability to survive in waterlogged soil conditions. This study used a genome-wide association study (GWAS) approach with 90K single nucleotide polymorphisms (SNPs) in a panel of 329 wheat genotypes, to reveal quantitative trait loci (QTL) conferring ARs and RCA. The wheat genotypes exposed to waterlogging were evaluated for ARs and RCA in both field and glasshouse over two consecutive years. Six and five significant marker-trait associations (MTAs) were identified for ARs and RCA formation under waterlogging, respectively. The most significant MTA for AR and RCA was found on chromosome 4B. Two wheat cultivars with contrasting waterlogging tolerance (tolerant: H-242, sensitive: H-195) were chosen to compare the development and regulation of aerenchyma in waterlogged conditions using staining methods. Results showed that under waterlogging conditions, H_2_O_2_ signal generated before aerenchyma formation in both sensitive and tolerant varieties with the tolerant variety accumulating more H_2_O_2_ and in a quicker manner compared to the sensitive one. Several genotypes which performed consistently well under different conditions can be used in breeding programs to develop waterlogging-tolerant wheat varieties.

## Introduction

Waterlogging is a major constraint to the production of winter wheat (*Triticum aestivum* L.) worldwide such as in the Yangtze River valley of China, the USA, Australia, Europe and Japan. Waterlogging occurs due to intermittent rainfall, irrigation practices, and/or poor soil drainage (Liu and Bassham, 2012; Manik et al., 2019). Waterlogging is deleterious to the growth and yield of wheat crops (Li et al., 2001; Setter and Waters, 2003). In Australia, about one million ha of wheat cropping area is affected by waterlogging, which causes 20%-50% of yield losses (Manik et al., 2019). The principal cause of damage to plants under waterlogging/saturated soil is due to inadequate oxygen supply since gas diffusion in water is tremendously slower than that in the air (Loreti et al., 2016).

Plants have developed several adaptive metabolic strategies to cope with oxygen deprivation under hypoxia/anoxic (Bailey-Serres et al., 2012; Voesenek and Bailey-Serres, 2013; Abbas et al., 2015). These include anatomical changes such as the formation of a barrier against radial oxygen loss, the development of adventitious roots (ARs) and root cortical aerenchyma (RCA) (Armstrong, 1971; Malik et al., 2003; Vidoz et al., 2010; Nelson et al, 2018; Zhang et al., 2015; Kim et al., 2015; Pan et al., 2019). The importance of AR in alleviating waterlogging stress has been observed in rice (*Oryza Sativa*; Lorbiecke and Sauter, 1999), *Rumex* spp. (Visser et al., 1996), tamarack (*Larix laricina*; Calvo-Polanco et al., 2012), *Eucalyptus* spp. (Argus et al., 2015), and tomato (*Solanum lycopersicum*). Adventitious roots enhance gas exchange, and water/nutrient uptake, thus contributing to plants’ survival under waterlogging (Steffens and Rasmussen, 2016). Root cortical aerenchyma, a special plant tissue containing enlarged gas space, facilitates oxygen delivery from above ground to root zones thus maintaining a sustainable root growth rate under hypoxia (Armstrong, 1979; Evans, 2003). Positive correlations between the proportion/area of aerenchyma and waterlogging tolerance have been witnessed in multiple crops, such as oats (*Avena sativa* L), triticale, wheat, cotton (*Anemone vitifolia Buch*), and barley (Pan et al., 2019; Thomson et al., 1992; Huang et al., 1994; Setter and Waters, 2003; Zhang et al., 2015). There are two types of aerenchyma according to the developmental process: lysigeny and schizogeny (Evans, 2003; Haque et al., 2010). Lysigenous aerenchyma is generally referred to as the aerenchyma developed in non-wetland species, such as wheat (Trought and Drew, 1980), barley (Benjamin and Greenway, 1979), and maize (Drew et al., 1979), when exposed to low oxygen environments. Schizogenous aerenchyma is generally the constitutive aerenchyma, formed by the separation of cells and growth patterns without cell death taking place. Schizogenous aerenchyma occurs in many species under non-waterlogged conditions, though in some species it can be increased in extent in waterlogged conditions (Evans, 2003).

Many other different agronomic and physiological traits have been used as indicators for plant waterlogging tolerance. These include leaf chlorosis (Li et al., 2008), ion toxicity (Pang et al., 2007; Huang et al., 2018), survival rate, germination rate index, leaf chlorophyll content, plant height index, dry matter weight (Yu and Chen, 2013), ROS formation (Gill et al., 2019), membrane potential maintenance (Gill et al., 2017), K^+^ retention in barley roots (Zeng et al., 2014). Using these traits, many QTL for waterlogging tolerance have been reported in barley (Li et al., 2008; Zhou, 2011; Broughton et al., 2015; Manik et al., 2022b), wheat (Yu et al., 2014; Ballesteros et al., 2015), maize (Qiu et al., 2007; Campbell et al., 2015) and other crops. Among the major QTL for wheat waterlogging tolerance, the one on chromosome 7A explained 23.92% of the phenotypic variance of germination rate index (Yu et al., 2014), and another QTL on chromosome 1BL explained 14 - 32% of phenotypic variation in chlorophyll and root/shoot dry matter under waterlogged conditions (Ballesteros et al., 2015). From a cross between a winter spelta (*T. spelta* L) and a winter wheat, more than 10 QTL associated with seedling survival and seedling growth index under waterlogging have been identified on different chromosomes with spelta providing most QTL for waterlogging tolerance (Burgos et al., 2001).

Fast formation of aerenchyma in adventitious roots is critical for root survival and waterlogging tolerance in cereal crops under waterlogged conditions (Zhang et al., 2016). However, there are limited reports on QTL for RCA and ARs, or the genetic control of waterlogging tolerance in wheat when compared to the significant amount of literature on other crops, such as rice (Toojinda et al., 2003), barley (Zhu et al, 2008; Manik et al., 2022b), soybean (Cornelious et al., 2005) and maize (Qiu et al., 2007). GWAS could be conducted using a diverse range of varieties without the development of a population (Buckler and Thornsberry, 2002). By using a large number of varieties, it facilitates the exploration of more genetic variations, including those with small effects and allelic diversity (Zhu et al., 2008; Juliana et al., 2018). Moreover, GWAS provides higher resolution mapping for identifying candidate genes (Nelson et al., 2018). The aim of this study was to link two-year phenotyping data from both the field and glasshouse with genotyping data using GWAS, therefore identifying potential QTL and searching for candidate genes associated with adventitious roots, cortical aerenchyma and root survival in wheat under waterlogging conditions.

## Materials and methods

### Plant materials and genotyping

A set of 335 wheat genotypes collected worldwide were used to evaluate their waterlogging tolerance. All these genotypes were obtained from China and Australian Grains GenBank. Genotyping of these accessions has been described in Kang et al. (2020) and Choudhury et al. (2019). Briefly, all accessions were genotyped using the Illumina iSelect 90,000 SNP bead chip assay described in Wei et al, 2019. A total of 38,379 SNPs were identified to be polymorphic in the population. For quality control, markers with a less than 90% call rate across samples, a minor allele frequency less than 0.05, or that were redundant were removed. Finally, 4,560 markers were selected for marker trait association analysis.

### Growth conditions, waterlogging treatment and assessment for aerenchyma formation and root survival

**Figure 1:**
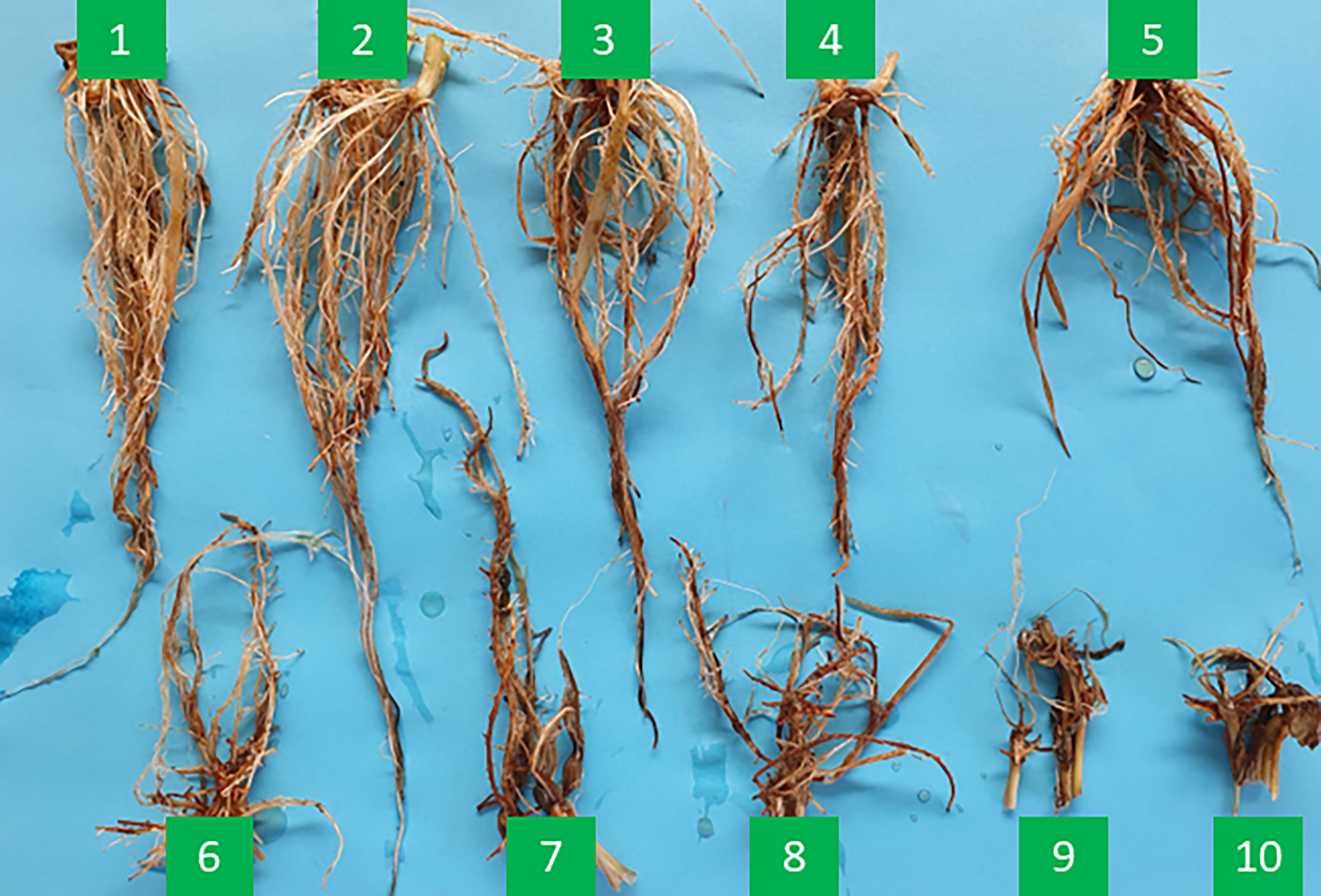
Root damage scores under waterlogging conditions. Damage score of 1: highly tolerant; damage score of 10: severely damaged with no survived roots.

Two glasshouse tank trials and one outdoor tank trial were undertaken at the University of Tasmania, Australia in 2021. For the experiment in the glasshouse, plants were grown in 60 cm × 120 cm × 60 cm bins, filled with soil from the field site prone to waterlogging. The outside tank experiment was performed in big cement tanks (120 cm x 240 cm x 70 cm), also filled with soil from the site prone to waterlogging. For all the glasshouse and tank experiments, waterlogging treatment was imposed at the 2-3 leaf stage. For the waterlogging treatment, the water was maintained at the soil surface level. The experiment was repeated three times in all the trials. Root samples were harvested when the roots of the most sensitive genotypes died (two weeks after waterlogging in glasshouse tank experiments and eight weeks after waterlogging in the outdoor tank experiment). Root survival was scored from 1 to 10 after terminating waterlogging treatment (**Figure 1**) with the most tolerant varieties being scored 1 (well-developed ARs and no dead roots) and the most sensitive/intolerant plants being scored 10 (all roots died) based on the methods by (Manik et al., 2022b). Aerenchyma formation was detected based on the method by Zhang et al. (2016). Root cortical aerenchyma formation in adventitious roots was checked 10 days after waterlogging. Approximately 2 cm long root segments were taken from the mature zone which was about 6 cm from the root apex. Cross sections were cut manually using razor blades and photographed under a bright field light microscope (Olympus BXBX53, Japan). The proportion of aerenchyma was visually scored based on digital images and the criteria are as followed: 0 = no aerenchyma, 4 = well-formed aerenchyma (Zhang et al., 2016).

### Genome-wide association analysis

For population correction, PCA (principal component analysis) was calculated using SNPRelate package as described in the previous publication (Kang et al., 2020), the optimum number of PCs (Principal components) was fixed in association models. GWAS was conducted using the rMVP package of R as described earlier (Yin et al., 2021; Manik et al., 2022b). In the association study, the notional p-value threshold to indicate a significant MTA was set to -Log10(p) ≥ 3.5.

In our study, GLM, MLM, and FarmCPU models were used for analysing the data on adventitious root development and root cortical aerenchyma formation scored from the 329 accessions and 18,132 SNPs. The best model was selected based on quantile-quantile (Q-Q) plots (**Figure S2**). The outcome variable in each model was the average of AR or RCA values from different trials.

The FarmCPU model was found to be the most suitable one which had a small number of extreme p-values. The FarmCPU method iteratively performs marker tests with pseudo-quantitative trait nucleotides (QTNs) as covariates in a fixed-effect model and keeps optimizing pseudo-QTNs in a random-effect model, and this process continues until no new pseudo QTNs are added (Liu et al., 2016). The joint capabilities of removing confounding effects between testing markers and kinship, preventing model overfitting, and controlling false positives have made the FarmCPU method one of the most efficient methods in modern GWAS (Knoch et al., 2020). Within our implementation of the rMVP model we used the following settings: default imputation, no principal components were adduced, “max Loop” was set to 10 (this exceeded what was required for our data), and the internal “FaST-LMM” method was used. Significant markers were visualized with a Manhattan plot and important p-value distributions from all the GLM, MLM and FarmCPU results (expected vs. observed p-values on a -log10 scale) were shown with Q-Q plots.

### Estimation of allelic effects on waterlogging tolerance

The allele at the locus of the detected SNPs responsible for increasing waterlogging tolerant (lower visual scoring (healthier roots or greater proportion of RCA)) is referred to as a “positive allele (T or TT)” while the allele associated with increasing waterlogging sensitivity was classified as a “negative allele (S or SS)”. The effects of SNPs were determined based on average matching scores of positive alleles and those of negative alleles.

### Candidate gene associated with waterlogging tolerance

SNPs at a level of -Log10(p) ≥ 5.5 were considered highly significant and were used to identify candidate genes. We used the physical chromosome positions and sequence of significant SNPs within the LD interval to identify the annotation of the high-confidence (HC) candidate genes. Based on the physical position of these SNPs (through blasting wheat genome on gramene.org), wheat gene IDs located either up or down 1 Mb of the physical position were searched for functional annotation. Gene functional annotation was obtained from the public database of wheat genome from 1 Kb (https://db.cngb.org/onekp/). Candidate genes with a possible connection to waterlogging tolerance were taken into consideration with high confidence.

### Statistical analysis and heritability

Phenotypic traits were summarised with descriptive statistics (mean and standard deviation).

### Histological staining

Aerenchyma formation follows programmed cell death (PCD) induced by histological metabolic changes, typically, the production of H_2_O_2_ under waterlogging (Cheng et al., 2016). To confirm this, the adventitious roots of wheat seedlings were sampled 1, 3, 5, and 7 d after waterlogging to examine aerenchyma development. Aerenchyma formation of wheat roots after 1 d, 3 d, 5 d, and 7 d waterlogging treatment was scored according to Zhang et al. (2016). Root cross sections at 40 mm above the root tip were sampled to study the characteristics of cells that develop aerenchyma using light micrographs. We used three pairs of wheat varieties showing different waterlogging tolerance (tolerant, AUS19402, H-242, KZYL-SARK, and sensitive, H-195, YU-1, H-197) for cell acidification staining and diaminobenzidine (DAB) staining. Among these pairs, H-242 and H-195 showed the greatest differences thus being selected for further studies.

DAB staining was performed as described previously (Xu et al., 2019). Briefly, cross sections of wheat roots sampled after 1 d, 2 d, 3 d and 7 d waterlogging treatment were incubated in 1 mg/ml DAB solution (pH = 5.5) for 1 h and then immersed in 75% ethanol. The localization of H_2_O_2_ signal (indicated by the pink or brown color) was monitored under a light microscope (Leica M205C, Germany).

To study cell acidification, the root cross sections sampled after 1, 2, 3 and 7 d waterlogging treatment were stained with 1% neutral red in 10 mM Tris-HCl (pH 7.0) for 5 s. Then the sections were placed in the petri dish with clean water and washed for 3 times. Excess water was removed and photos were taken under a light microscope (Xu et al., 2013).

## Results

### Waterlogging tolerance of wheat accessions

**Figure 2:**
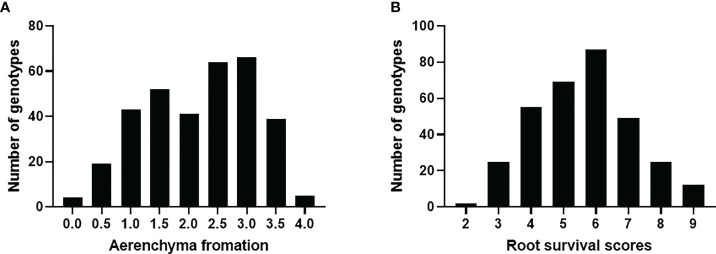
Distribution of aerenchyma formation **(A)** and root survival under waterlogging condition **(B)**.

The frequency distributions of root survival scores and aerenchyma formation of the 329 accessions are presented in **Figure 2**. Most of the genotypes formed aerenchyma with only 25 having the aerenchyma score of < 1 (**Figure 2A**). For root survival, most of the germplasm showed medium tolerance to waterlogging with less than 10% showing damage scores of < 3 and > 8, respectively (**Figure 2B**). The broad-sense heritability (h_B_^2^) for root survival was 0.37. Among those tolerant lines, Afghanistan 7, H-023, H-054, H-056, H-074, H-114, H-115, H-119, H-197, and Yu-03 showed consistently better root survival in both glasshouse trials and the tank trial while the roots of B-T-35, B-T-38, Bukovinka, CAZ53, H-083, H-249, H-251, H-252, IG43428, and Surhak 5688 died in all trials. Surprisingly, aerenchyma formation under waterlogging stress showed no significant correlation with root survival (R^2^ = 0.008, **Figure S1**). Some genotypes such as H-074 had no aerenchyma (aerenchyma score = 0.67) but showed good root survival, while some genotypes such as IG43428 and Surhak 5688 had aerenchyma scores of >3 but no roots survived. Detailed genotypic, phenotypic data and genetic map for the 329 common wheats are provided in **Supplementary Tables S1–S3**.

### Genome wide association study in wheat under waterlogging conditions

We use the same population as Kang et al. (2020), and the effect of population structure in our study was not significant. Three different GWAS models including generalised linear models (GLM), mixed linear models with random effects (MLM) and FarmCPU models were initially used and compared. The Q-Q plots from the three models were shown in **Figure S2**. FarmCPU was shown to be the best fitted model and thus we focused on the SNPs identified from the Farm CPU modelling.

**Figure 3:**
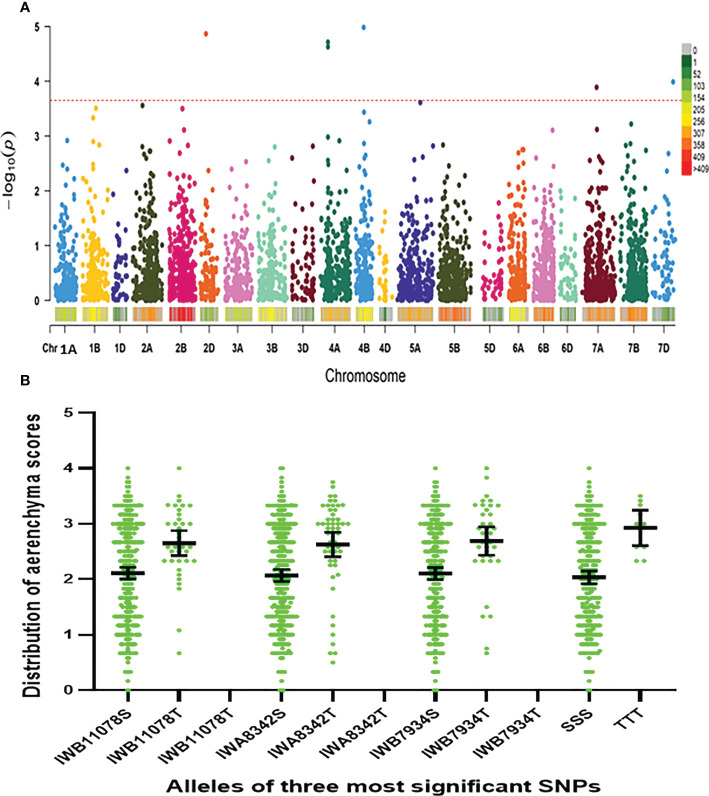
Marker-trait (aerenchyma formation) associations. **(A)**: GWAS aerenchyma formation using FarmCPU: Lod values of all the markers; **(B)**: distribution of aerenchyma formation of genotypes with three markers significantly correlated with aerenchyma formation and the combination of all three markers. T, tolerance allele; S, sensitive allele; TTT, tolerance alleles of all three markers; SSS, sensitive alleles of all three markers.

**Figure 4:**
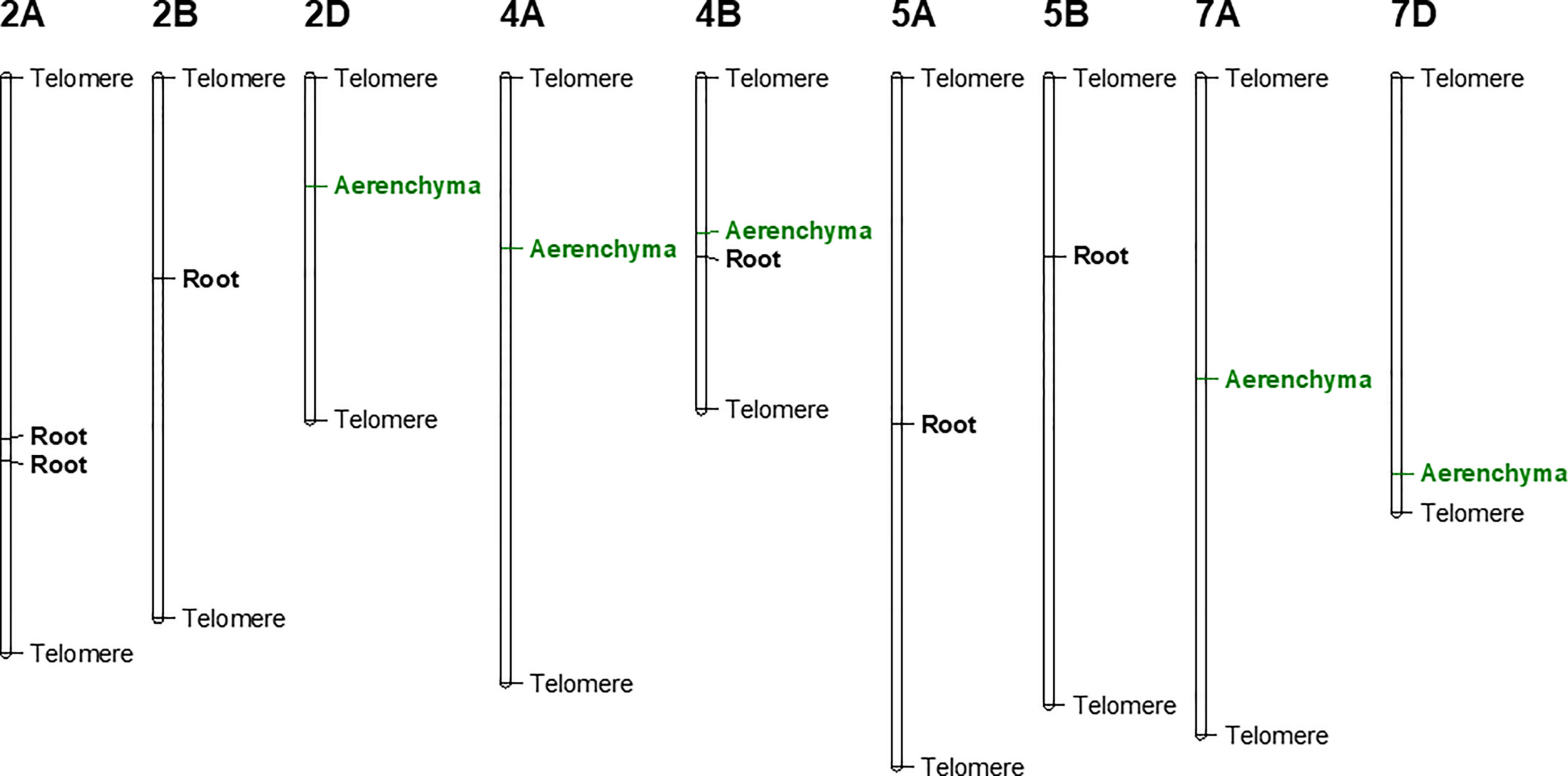
Genetic linkage map of identified MTAs. Root: markers associated with root survival under waterlogging condition; Aerenchyma: markers associated with aerenchyma formation under waterlogging condition.

Using the Farm CPU model, five significant MTAs were identified based on average scores of roots cortical aerenchyma (**Figure 3A**). The threshold for selecting the significant QTL is -log10(p) is equal or larger than 3.8. These MTAs were located on chromosomes 2D, 4A, 4B, 7A, and 7D (**Table 1**; **Figures 3A**, **Figure 4**). SNPIWB11078, SNPIWA8342 and SNPIWB7934 were the most significant MTAs. Overall, genotypes with the tolerance alleles (marked with T) showed a significantly higher proportion of aerenchyma than those with sensitive alleles (marked with S). These alleles had additive effects and all genotypes with three tolerant alleles had a greatest proportion of RCA (**Figure 3B**).

**Table 1:** QTL and associated markers for different traits.

Associated markers	Chromosome	Physical position(Mb)	Additive effect		Marker significance level	R^2^	minor allele frequency (MAF)
Root survival under waterlogging stress							
IWA2092	2A	388.71		0.69	1.0E-04	0.011	0.25
IWB11193	2A	413.64		0.55	8.9E-05	0.026	0.16
IWA3325	4B	193.52		0.73	4.1E-05	0.036	0.29
IWB22137	5A	374.32		-0.64	1.0E-04	0.013	0.22
IWB10994	5B	54.30		-0.61	7.2E-05	0.098	0.40
Aerenchyma formation under waterlogging stress							
IWB11078	2D	117.32		-0.59	1.4E-05	0.035	0.11
IWA8342	4A	185.89		0.50	2.4E-05	0.052	0.16
IWB41008	4A	185.89		-0.45	1.9E-05	0.043	0.17
IWB7934	4B	168.86		-0.53	1.0E-05	0.042	0.11
IWB33938	7A	325.39		-0.29	1.3E-04	0.029	0.50
IWB16121	7D	427.90		-0.43	1.0E-04	0.014	0.18

**Figure 5:**
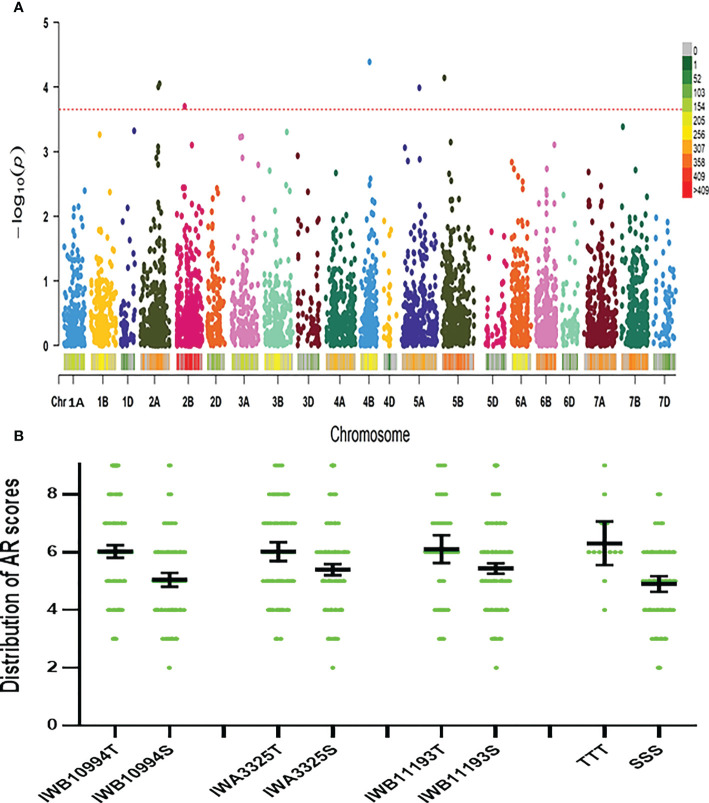
Marker-trait (root survival) associations. **(A)**: GWAS for root survival using FarmCPU: Lod values of all the markers; **(B)**: distribution of root survival scores of genotypes with three markers significantly correlated with root survival and the combination of all three markers. T, tolerance allele; S, sensitive allele; TTT, tolerance alleles of all three markers; SSS, sensitive alleles of all three markers.

Six significant MTAs were identified based on mean root survival scores, and these MTAs were located on chromosomes 2A (two), 2B, 4B, 5A and 5B (**Figures 4**, **5A**). The genotypes with tolerance alleles (marked with T) showed significantly better root survival than those with sensitive alleles (marked with S) and the combination of all three different tolerance alleles also improved root survival (**Figure 5B**). Among all the MTAs, only one MTA for root survival on 4B was located very close to one MTA for aerenchyma formation, confirming the non-significant correlation between aerenchyma formation and root survival.

### Histological staining during aerenchyma formation

**Figure 6:**
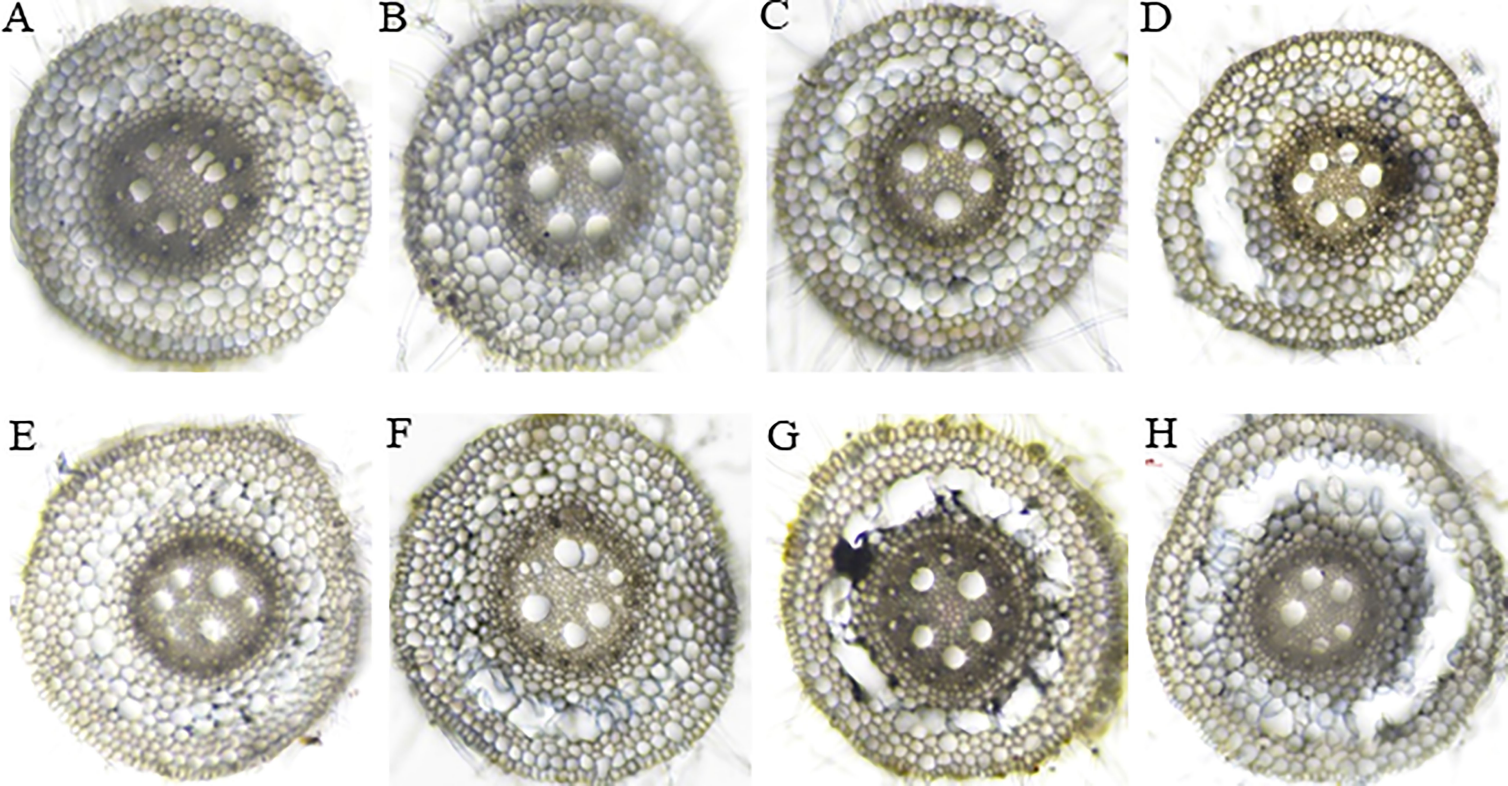
Aerenchyma formation in the root cross-section sampled at 4 cm from the root tips in wheat genotypes with different degrees of aerenchyma formation after waterlogging treatment. Upper panel: H-195; Bottom panel: H-242. **(A, E)**, **(B, F)**, **(C, G)**, and **(D, H)**: waterlogging for 1, 3, 5 and 7 days, respectively.

**Figure 7:**
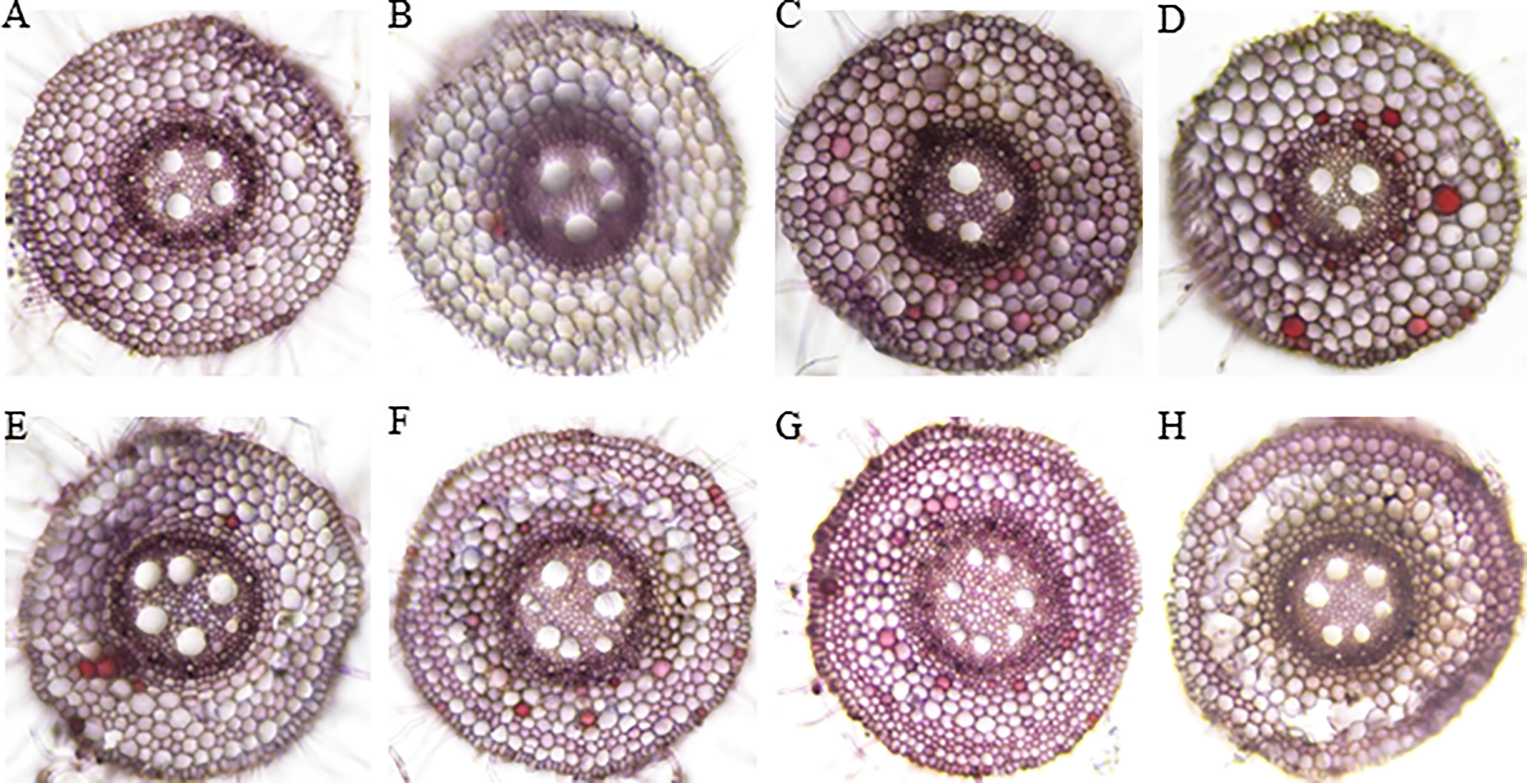
Neutral red staining for cell acidification. Upper panel: H-195; Bottom panel: H-242. **(A, E)**, **(B, F)**, **(C, G)**, and **(D, H)**: waterlogging for 1 to 4 d, respectively.

**Figure 8:**
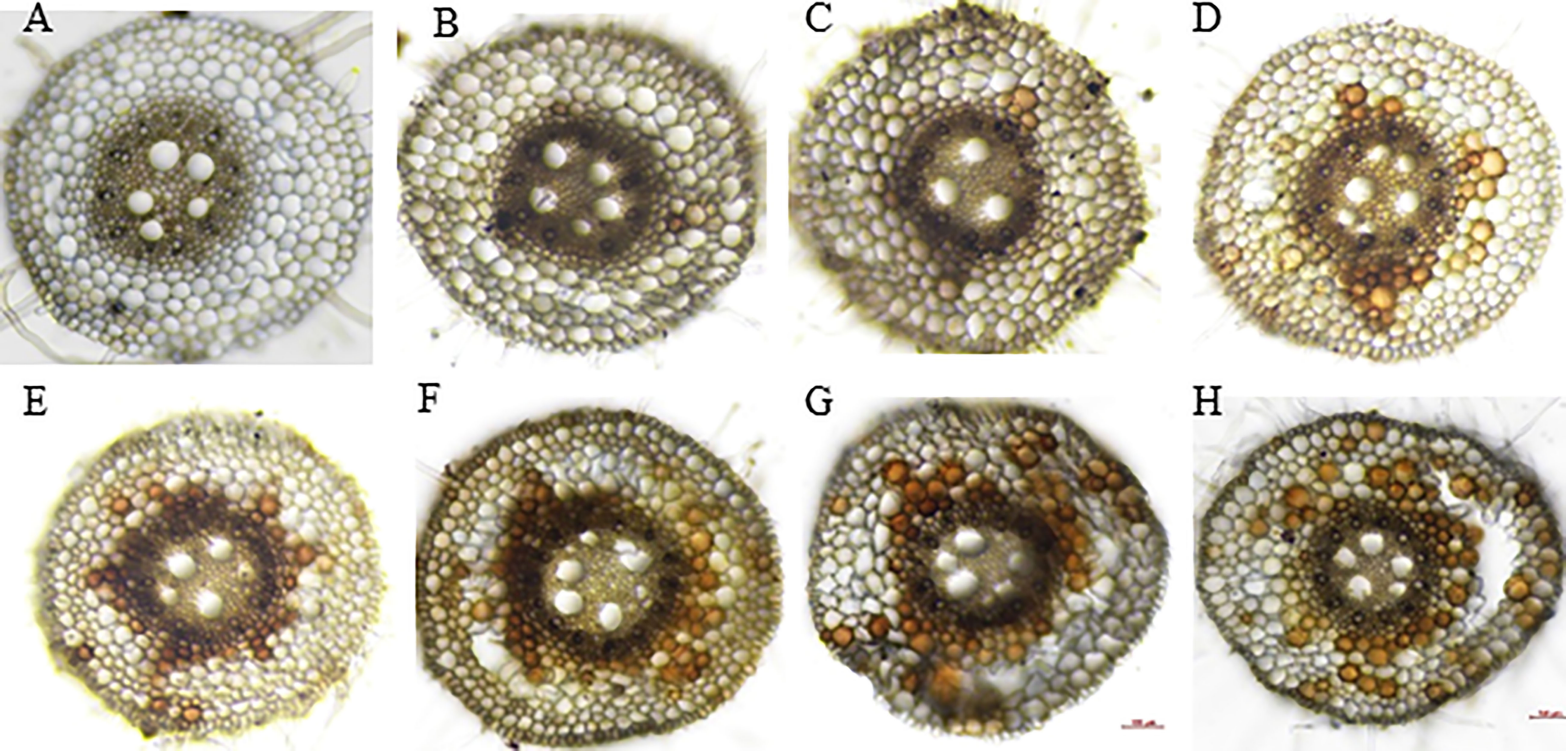
DAB staining for H_2_O_2_ signal. Upper panel: H-195; Bottom panel: H-242. **(A, E)**, **(B, F)**, **(C, G)** and **(D, H)**: waterlogging for 1 to 4 days, respectively.

The root cross-section taken at 4 cm from the root tips showed that the root cells remained intact thus no aerenchyma was formed even after five days of waterlogging treatment in sensitive variety H-195 (**Figure 6**). In tolerant variety H-242, aerenchyma formation started on the third day after waterlogging (**Figure 6**), and ~70% and >80% of the root cross-section area consisted of aerenchyma on the fifth and seventh day after waterlogging, respectively. This indicated an earlier formation of aerenchyma and larger proportion of aerenchyma in root cross-section of H-242 relative to H-195 (**Figure 6**). Evidence regarding the programmed root cell death was witnessed from the neutral red staining which similarly reflects the cell death scenario (**Figure 7**). Root cross-section of H-242 showed a higher accumulation of neutral staining, suggesting that a higher proportion of root cells were dying during waterlogging, consistent with the mechanism of aerenchyma formation against waterlogging in wheat (**Figure 7**). To further confirm whether reactive oxygen species (ROS) was involved in aerenchyma formation (PCD induced), histological staining was applied to monitor H_2_O_2_ production in the root cross-sections on selected representative waterlogging days. H_2_O_2_ was abundantly detected in the central cortical cells (the pre-aerenchyma cells) and was also seen in the vasculature with tolerant variety H-242 showing quicker response and accumulating more H_2_O_2_ than sensitive variety H-195 (**Figure 8**). All these observations indicated that under waterlogging, the waterlogging tolerant variety employs the PCD process to produce aerenchyma induced by H_2_O_2_.

## Discussion

### QTL for root traits associated with wheat waterlogging tolerance

Very few studies have investigated root physiological or morphological traits to identify QTL associated with waterlogging tolerance in wheat due to the difficulty of phenotyping. Instead, leaf physiological traits have been more often used for assessing wheat waterlogging tolerance. For example, by assessing leaf chlorosis at the maturity stage of wheat crops utilizing the SC (SHWL1 × Chuanmai 32) wheat population, 10 QTL were identified responsible for waterlogging tolerance (Yu and Chen, 2013). Furthermore, Yu and Chen (2013) assessed waterlogging tolerance using the wheat ITMI population W7984/Opata85 at the early growth stage, and found 36 QTL associated with aboveground traits (including survival rate, germination rate index, leaf chlorophyll content, plant height index, dry matter weight) on 18 chromosomes. Burgos et al. (2001) found that QTL associated with seedling survival/seedling growth index under waterlogging are on chromosomes 2A, 2B, 3A, 3B, 4B, 5A, 6A and 7S which explained 35.5% of the phenotypic variance (Burgos et al., 2001). Using recombinant inbred lines (RILs) derived from USG3209 × Jaypee, 48 QTL clustering into 10 genomic regions were consistently detected both in greenhouse and field trials, among which three QTL were identified on chromosome 1BL, explaining 22-32% phenotypic variance, and a major QTL (*QSpad3.ua-1D.5*) for chlorophyll content on chromosome 1D explained 24% phenotypic variance under environment-controlled greenhouse conditions (Ballesteros et al., 2015). However, none of those authors have investigated root aerenchyma formation in response to waterlogging.

Our findings offer a strong explanation that the variations in leaf survival performance is highly due to the difference in root aerenchyma development under waterlogging. MTAs for root survival performance were identified on chromosomes 2B and 5A in our study, which were consistent with results of Burgos et al. (2001). This highlights the possible linkage between root aerenchyma development and leaf growth under waterlogging. Besides, a major QTL on chromosome 7A associated with germination rate index was found under waterlogging conditions, explaining 23.92% of phenotypic variance in wheat (Yu et al., 2014). This QTL is located at a close position to the MTA for aerenchyma formation on 7A from our study (**Figure 4**). However, most of the QTL for root traits identified in our study were different from the previously reported QTL in wheat waterlogging utilizing leaf physiological traits (Tong et al., 2021). Therefore, our study case offers novelty in finding specific root survival related QTL in wheat responsible for waterlogging tolerance.

## Candidate genes

Based on the physical positions of markers that were significantly linked with those identified QTL, we further investigated candidate genes by investigating the annotated genes within these zones ( ± 1 cM from the maker position). Annotated genes functioning in ROS production, Ca^2+^ binding, and ethylene metabolism were highly regarded as potential candidate genes. Therefore, a total of 38 annotated wheat genes were identified from the proposed QTL in this study (**Table S1**). Of which, eight genes were highlighted as the potential candidate genes associated with ROS production according to annotated gene functions. ROS plays an important role in inducing PCD for aerenchyma development (Cheng et al., 2016). Within the QTL on chromosome 4B, gene *TraesCS4B02G176900* (encodes peroxidase) was co-located in the same zone, indicating that ROS metabolism is potentially involved in wheat waterlogging tolerance. *TraesCS2B02G229100* (encodes detoxification protein) correlates with the QTL on chromosome 2B and is potentially involved in the degradation of ROS under waterlogging (Li et al., 2018). Another gene *TraesCS2B02G230100* (encodes Histone H4) within the same QTL could also be a potential candidate gene. Direct evidence has been supplied that histone maintains a role in deciding cell wall degradation and thus participating in aerenchyma formation in wheat seminal roots (Li et al., 2019) under waterlogging. *TraesCS4A02G067800* encoding histone located on 4A is also a candidate gene (**Table S1**).

Ethylene response factor (ethylene related signaling components) is a representative regulator for crop waterlogging tolerance. Through maintaining the constitutive expression of ERFVII (group VII ethylene response factor) in transgenic wheat plants, waterlogging tolerance is significantly enhanced without grain yield penalty (Wei et al., 2019). In the present study, *TraesCS5B02G214400* (directly encoding ethylene responsive factor 5a) linked with the QTL on 5B was a most likely candidate gene. Besides, hormonal regulation under waterlogging is one of the mechanisms that plants utilize to fight against waterlogging (Nguyen et al., 2018). ABA biosynthesis genes, such as NCED1 and NCED2, are important in wheat waterlogging tolerance (Nguyen et al., 2018). Thus ABI3 (ABA insensitive 3, ABI interacting protein) encoded by *TraesCS5B02G214500* (within the QTL on 5B) was a potential candidate gene. Alanine aminotransferase links glycolysis and TCA cycle (tricarboxylic acid cycle) under hypoxia induced by waterlogging (Rocha et al., 2010), thus, *TraesCS5B02G214700* on 2D (encodes alanine aminotransferase) could contribute to RCA QTL on 2D (**Table S1**).

## Mechanisms for aerenchyma formation

Histochemical staining results in our study aligned with the mechanism of ROS-induced PCD for aerenchyma formation. Consistent with their findings of Xu et al. (2013) showing that ROS accumulation starts in the mid-cortex cells along with the development of aerenchyma in wheat, our results indicated that early cortical cell death started from the mid-cortex (**Figures 6**, **7**) along with the development of aerenchyma **(** **Figure 6** **)**. The neutral red staining under waterlogging conditions further illustrated the dying cell (**Figure 7**). Furthermore, lysigenous aerenchyma formation in wheat was reported to be regulated through ROS signaling (Yamauchi et al., 2014), further indicating the strong links between ROS production and aerenchyma formation. In our study, ROS accumulation under waterlogging was evidenced by DAB staining (for H_2_O_2_ detection) (**Figure 8**). H_2_O_2_ was abundantly detected (reflected by the brown precipitate) in the central cortical cells (pre-aerenchymal cells) (**Figure 8**). Our results suggested that aerenchyma development in wheat roots began in the central cortical cells (pre-aerenchymal cells) with further expansion to its surrounding cells, consistent with the previous report (Xu et al., 2013). Moreover, DAB staining also indicated the strong involvement of H_2_O_2_ induced PCD during aerenchyma formation (Xu et al., 2013). A similar mechanism has been confirmed by Steffens and Sauter (2005) and Steffens et al. (2011), showing that H_2_O_2_ is required for ethylene-induced epidermal death which is responsible for aerenchyma development in rice stems.

### The relationship between aerenchyma formation and root survival

Our results showed significant variation in root survival in response to waterlogging among 329 wheat genotypes with diverse genetic background, however, there was lack of correlation between root survival and aerenchyma formation in the present study, indicating that multiple mechanisms underlying root survival are involved.

Many studies show that aerenchyma formation and root survival are highly related (Thomson et al., 1990; Haque et al., 2012), and the aerenchyma formation greatly contributes to the survival of root system (Thomson et al., 1990; Haque et al., 2012). For example, newly produced adventitious roots are adapted with more aerenchyma than seminal roots in waterlogged soil or O_2_-deficient solutions, along with consecutive root elongation under saturated conditions (Thomson et al., 1992; Malik et al., 2002). In our study, fast aerenchyma development was observed in tolerant variety H-242 under waterlogging, along with the highly induced H_2_O_2_ accumulation (**Figure 6**) and greater root cell death (**Figure 7**), suggesting that root aerenchyma formation is involved in wheat waterlogging tolerance. These observations highly aligned with the classic defense mechanism that waterlogging triggers reactive oxygen species production which led to PCD for root aerenchyma formation under waterlogging (Cheng et al., 2016). Other than aerenchyma formation, other alternative waterlogging tolerance mechanisms were also reported. For example, root architecture was found to contribute to waterlogging tolerance (Haque et al., 2012). Root architecture involves axile and surface adventitious roots development induced by salicylic acid (SA). The reduction in redox potential due to waterlogging caused accumulation of toxin ions and secondary metabolites, which inhibit plant growth under waterlogging, skeletons as another defense mechanism. It was found that barley genotypes with better tolerance to Mn^2+^ or Fe^2+^ toxicity perform better in waterlogging soil (Pang et al., 2007; Hossein and Rengel, 2010; Hossein et al., 2012). Similarly, some barley genotypes with no RCA also show a degree of waterlogging tolerance (Manik et al., 2022a), suggesting that the tolerance to toxicity contributes to waterlogging resistance. In wheat, varieties tolerant to ion toxicities usually grow better under waterlogging-affected acid soil, disclosing the contribution of resistance to toxic compounds under waterlogging (Hossein and Rengel, 2010; Hossein et al., 2012). However, root aerenchyma development under waterlogging still serves as the primary mechanism for defending which involves ethylene biosynthesis (Koramutla et al., 2022). Barley varieties having QTL associated with root cortical aerenchyma showed better waterlogging tolerance, reflected by the higher aerenchyma area, more white (healthy and functional) adventitious roots, higher shoot biomass (Manik et al., 2022a). QTL responsible for root survival and root aerenchyma development identified in our study (**Figure 4**) provide multiple mechanisms that can be were utilized by crops while under waterlogging.

### Factors influence the assessment of waterlogging tolerance and the development of aerenchyma

Factors affecting the assessment of waterlogging can be simply categorized into underground and aboveground. Leaf traits aboveground for assessing waterlogging damage are mainly utilized for QTL detection in wheat (Burgos et al., 2001; Yu and Chen, 2013). However, given the hypoxia environment (induced by waterlogging) in soil that initially limited/affected root growth due to oxygen deficiency (or soil toxicity), root damage may have occurred at early waterlogging stress stage before leaf related traits become affected. Therefore, only focusing on leaf related traits for waterlogging QTL detections has limitations. In our research, we primarily investigated root survival and root aerenchyma development under wheat waterlogging stress, which identified the QTL for root development under waterlogging (**Figure 4**).

Aerenchyma formation is determined by a combination of variable aspects. The porosity of adventitious root increases due to the formation of aerenchyma, which depends on genotype, plant developmental stage, duration of O_2_ deficiency, and treatment method (Thomson et al., 1990; Huang et al., 1997; Wiengweera et al., 1997; Malik et al., 2001; Malik et al., 2002; Manik et al., 2019; Tong et al, 2021). Genetic variation is the dominant factor that determines aerenchyma development under waterlogging, which is also the key basis for GWAS/QTL analysis. Previous research has illustrated the importance of root aerenchyma for waterlogging tolerance. For instance, waterlogging-tolerant genotypes always had higher root porosities than sensitive genotypes if growing in an N_2_-flushed nutrient solution for 20 days, demonstrating the importance of genotypic variation under waterlogging (Boru et al., 2003). Besides, plant growth stages differ in aerenchyma development. Seminal roots of young seedlings form aerenchyma whereas older seminal roots lose this capacity (Thomson et al., 1990; Xu et al., 2013; Tong et al., 2021). Our research not only confirmed the importance of aerenchyma formation in wheat waterlogging tolerance, but also agrees with previous research cases that the involvement of H_2_O_2_ induced PCD is critical for aerenchyma development (Xu et al., 2013; Cheng et al., 2016).

## Conclusions

Waterlogging seriously affected wheat root growth. GWAS analysis identified six and five MTAs/QTL in wheat associated with the formation of adventitious roots and aerenchyma formation, respectively, under waterlogging stress. Several genotypes consistently produced a large number of adventitious roots and formed a large proportion of aerenchyma in root cortical cells could be used in breeding programs to develop waterlogging tolerant wheat varieties. Through histochemical staining, our study confirmed that H_2_O_2_ induced PCD led to aerenchyma formation in wheat adventitious roots. Candidate genes for these QTL are most likely ROS, ethylene or ABA related regulatory elements. Our research also illustrated an alternative tolerance mechanism that may involve soil toxicity tolerance under waterlogged soil. Similarly, wheat lines which don’t have aerenchyma but indicated stable tolerance to waterlogging can be used to pyramid different waterlogging tolerance mechanisms. As healthy root development under waterlogging conditions is the ultimate waterlogging tolerance indicator, future research should concentrate more on roots and mechanisms for root survival and development under the hypoxia environment. The combination of QTL for aerenchyma formation and soil toxicity tolerance can be more effectively used to improve wheat waterlogging tolerance.

## Data availability statement

The data presented in the study are deposited in the Dryad repository, accession number doi:10.5061/dryad.wh70rxwrk.

## Author contributions

MZ acquired the funding, and participated in supervision; WZ participated in supervision, LX, YN, and CZ conducted the field trials and data collection and data analysis; LX, YN, and CZ carried out data visualization; HL collected the data; LX, MZ, and JP wrote, reviewed and edited the draft. All authors contributed to the article and approved the submitted version.

## Funding

This project is funded by the Grains Research and Development Corporation (GRDC) of Australia and Hubei Key Research and Development Program (2021BBA225).

## Conflict of interest

The authors declare that the research was conducted in the absence of any commercial or financial relationships that could be construed as a potential conflict of interest.

## Publisher’s note

All claims expressed in this article are solely those of the authors and do not necessarily represent those of their affiliated organizations, or those of the publisher, the editors and the reviewers. Any product that may be evaluated in this article, or claim that may be made by its manufacturer, is not guaranteed or endorsed by the publisher.

## References

[B1] Abbas M. Berckhan S. Rooney D. J. Gibbs D. J. Vicente C. J. Sousa C. C.. (2015). Oxygen sensing coordinates photomorphogenesis to facilitate seedling survival. Curr. Biol. 25, 1483–1488. doi: 10.1093/jxb/eraa442 25981794 PMC4454774

[B2] Argus R. E. Colmer T. D. Grierson P. F. (2015). Early physiological flood tolerance is followed by slow post-flooding root recovery in the dryland riparian tree *Eucalyptus camaldulensis subsp refulgens* . Plant Cell Environ. 36, 1189–1199. doi: 10.1111/pce.12473 25328049

[B3] Armstrong W. (1971). Radial oxygen losses from intact rice roots as affected by distance from the apex, respiration and water logging. Physiol. Plant 25, 192–197. doi: 10.1111/j.1399-3054.1971.tb01427.x

[B4] Armstrong W. D. (1979). Aeration in higher plants. Adv. Bot. Res. 7, 225–332. doi: 10.1016/S0065-2296(08)60089-0

[B5] Bailey-Serres J. Lee S. C. Brinton E. (2012). Waterproofing crops: Effective flooding survival strategies. Plant Physiol. 160, 1698–1709. doi: 10.2307/41812018 23093359 PMC3510103

[B6] Ballesteros D. C. Mason R. E. Addison C. K. Andrea Acuña M. Nelly Arguello M. Subramanian N.. (2015). Tolerance of wheat to vegetative stage soil waterlogging is conditioned by both constitutive and adaptive QTL. Euphytica. 201, 329–343. doi: 10.1007/s10681-014-1184-3

[B7] Benjamin L. R. Greenway H. (1979). Effects of a range of O_2_ concentrations on porosity of barley roots and on their sugar and protein concentrations. Ann. Bot. 43, 383–391. doi: 10.1093/oxfordjournals.aob.a085646

[B8] Boru G. van Ginkel M. Trethowan R. M. Boersma L. Kronstad W. E. (2003). Oxygen use from solution by wheat genotypes differing in tolerance to waterlogging. Euphytica. 132, 151–158. doi: 10.1023/A:1024622405505

[B9] Broughton S. Zhou G. F. Teakle L. N. Matsuda R. Zhou M. X. O’Leary A. R.. (2015). Waterlogging tolerance is associated with root porosity in barley (*Hordeum vulgare* l.). Mol. Breed. 35, 27.doi: 10.1007/s11032-015-0243-3

[B10] Buckler E. S. Thornsberry J. M. (2002). Plant molecular diversity and applications to genomics. Curt Opin. Plant Biol. 5, 107–111. doi: 10.1016/S1369-5266(02)00238-8 11856604

[B11] Burgos M. S. Messmer M. M. Stamp P. Schmid J. E. (2001). Flooding tolerance of spelt (*Triticum spelta* l.) compared to wheat (*Triticum aestivum* l.)- a physiological and genetic approach. Euphytica. 122, 287–295. doi: 10.1023/a:1012945902299

[B12] Calvo-Polanco M. Señorans J. Zwiazek J. J. (2012). Role of adventitious roots in water relations of tamarack (*Larix laricina*) seedlings exposed to flooding. BMC Plant Biol. 12, 99. doi: 10.1186/1471-2229-12-99 22738296 PMC3431261

[B13] Campbell M. T. Proctor C. A. Dou Y. Schmitz A. J. Phansak P. Kruger G. R.. (2015). Genetic and molecular characterization of submergence response identifies Subtol6 as a major submergence tolerance locus in maize. PloS One 10, e120385. doi: 10.1371/journal.pone.0120385 PMC4373911 25806518

[B14] Cheng X. X. Yu M. Zhang N. Zhou Z. Q. Xu Q. T. Mei Z. F.. (2016). Reactive oxygen species regulate programmed cell death progress of endosperm in winter wheat (*Triticum aestivum* l.) under waterlogging. Protoplasma. 253, 311–327. doi: 10.1007/s00709-015-0811-8 25854793

[B15] Choudhury S. Hu H. Fan Y. Larkin P. Hayden M. Forrest K.. (2019). Identification of new QTL contributing to barley yellow dwarf virus-PAV (BYDV-PAV) resistance in wheat. Plant Dis. 103 (11), 2798–2803. doi: 10.1094/PDIS-02-19-0271-RE 31524094

[B16] Cornelious B. Chen P. Chen Y. Leon N. Shannon J. G. Wang D. (2005). Identification of QTLs underlying water-logging tolerance in soybean. Mol. Breed. 16, 103–112. doi: 10.1007/s11032-005-5911-2

[B17] Drew M. C. Jackson M. B. Giffard S. (1979). Ethylene-promoted adventitious rooting and development of cortical air spaces (aerenchyma) in roots may be adaptive responses to flooding in *Zea mays* l. Planta. 147, 83–88. doi: 10.1007/BF00384595 24310899

[B18] Evans D. E. (2003). Aerenchyma formation. New Phytol. 161, 35–49. doi: 10.1046/j.1469-8137.2003.00907.x

[B19] Gill M. B. Zeng F. Shabala L. Zhang G. Fan Y. Shabala S.. (2017). Cell-based phenotyping reveals QTL for membrane potential maintenance associated with hypoxia and salinity stress tolerance in barley. Front. Plant Sci. 8. doi: 10.3389/fpls.2017.01941 PMC5696338 29201033

[B20] Gill M. B. Zeng F. Shabala L. Zhang G. Yu M. Demidchik V.. (2019). Identification of QTL related to ROS formation under hypoxia and their association with waterlogging and salt tolerance in barley. Int. J. Mol. Sci. 20, 699. doi: 10.3390/ijms20030699 30736310 PMC6387252

[B21] Haque M. E. Abe F. Kawaguchi K. (2010). Formation and extension of lysigenous aerenchyma in seminal root cortex of spring wheat (*Triticum aestivum* cv. bobwhite line SH 98 26) seedlings under different strengths of waterlogging. Plant Root. 4, 31–39. doi: 10.3117/plantroot.4.31

[B22] Haque M. E. Oyanagi A. Kawaguchi K. (2012). Aerenchyma formation in the seminal roots of Japanese wheat cultivars in relation to growth under waterlogged conditions. Plant Prod Sci. 15, 164–173. doi: 10.1626/pps.15.164

[B23] Hossein K. S. Barker S. J. Rengel Z. (2012). Tolerance to ion toxicities enhances wheat (*Triticum aestivum* l.) grain yield in waterlogged acidic soils. Plant Soil. 354, 371–381. doi: 10.1007/s11104-011-1073-7

[B24] Hossein K. S. Rengel Z. (2010). Aluminum, manganese, and iron tolerance improves performance of wheat genotypes in waterlogged acidic soils. J. Plant Nutr. Soil Sci. 173, 461–468. doi: 10.1002/jpln.200900316

[B25] Huang X. Fan Y. Shabala L. Rengel Z. Shabala S. Zhou M. X. (2018). A major QTL controlling the tolerance to manganese toxicity in barley (*Hordeum vulgare* l.). Mol. Breed 38, 16. doi: 10.1007/s11032-017-0767-9

[B26] Huang B. R. Johnson J. W. Box J. E. NeSmith D. S. (1997). Root characteristics and hormone activity of wheat in response to hypoxia and ethylene. Crop Sci. 37, 812–818. doi: 10.2135/cropsci1997.0011183X003700030020x

[B27] Huang B. R. Johnson J. W. Nesmith D. S. Bridges D. C. (1994). Root and shoot growth of wheat genotypes in response to hypoxia and subsequent resumption of aeration. Crop Sci. 34, 1538–1544. doi: 10.2135/cropsci1994.0011183X003400060023x

[B28] Juliana P. Singh R. P. Singh P. K. Poland J. A. Bergstrom G. C. Huerta-Espino J.. (2018). Genome-wide association mapping for resistance to leaf rust, stripe rust and tan spot in wheat reveals potential candidate genes. Theor. Appl. Genet. 131, 1405–1422. doi: 10.1007/s00122-018-3086-6 29589041 PMC6004277

[B29] Kang Y. Barry K. Cao F. Zhou M. (2020). Genome-wide association mapping for adult resistance to powdery mildew in common wheat. Mol. Biol. Rep. 47, 1241–1256. doi: 10.1007/s11033-019-05225-4 31813131

[B30] Kim Y. H. Hwang S. J. Waqas M. Khan A. L. Lee J. H. Lee J. D.. (2015). Comparative analysis of endogenous hormones level in two soybean (*Glycine max* l.) lines differing in waterlogging tolerance. Front. Plant Sci. 6. doi: 10.3389/fpls.2015.00714 PMC4585003 26442028

[B31] Knoch D. Abbadi A. Grandke F. Meyer R. C. Samans B. Werner C. R.. (2020). Strong temporal dynamics of QTL action on plant growth progression revealed through high-throughput phenotyping in canola. Plant Biotechnol. J. 18, 68–82. doi: 10.1111/pbi.13171 31125482 PMC6920335

[B32] Koramutla M. K. Tuan P. A. Ayele B. T. (2022). Salicylic acid enhances adventitious root and aerenchyma formation in wheat under waterlogged conditions. Int. J. Mol. Sci. 23, 1243. doi: 10.3390/ijms23031243 35163167 PMC8835647

[B33] Li J. C. Dong Q. Yu S. L. (2001). Effects of waterlogging at different growth stages on photosynthesis and yield of different wheat cultivars. Acta Agronomica Sinica. 27, 434–441. doi: 10.1136/bmj.2.6096.1220

[B34] Li C. Liu D. Lin Z. Guan B. Liu D. Yang L.. (2019). Histone acetylation modification affects cell wall degradation and aerenchyma formation in wheat seminal roots under waterlogging. Plant Growth Regul. 87, 149–163. doi: 10.1007/s10725-018-0460-y

[B35] Liu Y. Bassham D. C. (2012). Autophagy: pathways for self-eating in plant cells. Ann. Rev. Plant Biol. 63, 215–237. doi: 10.1146/annurev-arplant-042811-105441 22242963

[B36] Liu X. Huang M. Fan B. Buckler E. S. Zhang Z. (2016). Iterative usage of fixed and random effect models for powerful and efficient genome-wide association studies. PloS Genet. 12, e1005767. doi: 10.1371/journal.pgen.1005767 26828793 PMC4734661

[B37] Li H. Vaillancourt R. Mendham N. Zhou M. (2008). Comparative mapping of quantitative trait loci associated with waterlogging tolerance in barley (*Hordeum vulgare* l.). BMC Genom. 9, 401. doi: 10.1186/1471-2164-9-401 PMC2533678 18752688

[B38] Lorbiecke R. Sauter M. (1999). Adventitious root growth and cell cycle induction in deepwater rice. Plant Physiol. 119, 21–29. doi: 10.1104/pp.119.1.21 9880342 PMC32222

[B39] Loreti E. van Veen H. Perata P. (2016). Plant responses to flooding stress. Curr. Opin. Plant Biol. 33, 64–71. doi: 10.1016/j.pbi.2016.06.005 27322538

[B40] Malik A. I. Colmer T. D. Lambers H. Schortemeyer M. (2001). Changes in physiological and morphological traits of roots and shoots of wheat in response to different depths of waterlogging. Aus J. Plant Physiol. 28, 1121–1131. doi: 10.1016/S0014-5793(97)00086-0

[B41] Malik A. I. Colmer T. D. Lambers H. Schortemeyer M. (2003). Aerenchyma formation and radial O_2_ loss along adventitious roots of wheat with only the apical root portion exposed to O_2_ deficiency. Plant Cell Environ. 26, 1713–1722. doi: 10.1046/j.1365-3040.2003.01089.x

[B42] Malik A. I. Setter T. L. Colmer T. D. Lambers H. Schortemeyer M. (2002). Short-term waterlogging has long-term effects on the growth and physiology of wheat. New Phytol. 153, 225–236. doi: 10.2307/1513791

[B43] Manik S. M. N. Pengilley G. Dean G. Field B. Shabala S. Zhou M. (2019). Soil and crop management practices to minimize the impact of waterlogging on crop productivity. Front. Plant Sci. 10. doi: 10.3389/fpls.2019.00140 PMC6379354 30809241

[B44] Manik S. M. N. Quamruzzaman M. Livermore M. Zhao C. Johnson P. Hunt I.. (2022a). Impacts of barley root cortical aerenchyma on growth, physiology, yield components, and grain quality under field waterlogging conditions. Field Crops Res. 279, 108461. doi: 10.1016/j.fcr.2022.108461

[B45] Manik S. M. N. Quamruzzaman M. Zhao C. C. Johnson P. Hunt I. Shabala S.. (2022b). Genome-wide association study reveals marker trait associations (MTA) for waterlogging-triggered adventitious roots and aerenchyma formation in barley. Int. J. Mol. Sci. 23, 3341. doi: 10.3390/ijms23063341 35328762 PMC8954902

[B46] Nelson R. Wiesner-Hanks T. Wisser R. Balint-Kurti P. (2018). Navigating complexity to breed disease-resistant crops. Nat. Rev. Genet. 19, 21–33. doi: 10.1038/nrg.2017.82 29109524

[B47] Nguyen T. Tuan P. Mukherjee S. Son S. Ayele B. (2018). Hormonal regulation in adventitious roots and during their emergence under waterlogged conditions in wheat. J. Exp. Bot. 69 (16), 4065–4082. doi: 10.1093/jxb/ery190 29788353 PMC6054230

[B48] Pang J. Cuin T. A. Shabala L. Zhou M. Mendham N. Shabala S. (2007). Effect of secondary metabolites associated with anaerobic soil conditions on ion fluxes and electrophysiology in barley roots. Plant Physiol. 145, 266–276. doi: 10.1104/pp.107.102624 17660351 PMC1976565

[B49] Pan R. He D. Xu L. Zhou M. Li C. Wu C.. (2019). Proteomic analysis reveals response of differential wheat (*Triticum aestivum* l.) genotypes to oxygen deficiency stress. BMC Genomics 20, 60. doi: 10.1186/s12864-018-5405-3 30658567 PMC6339445

[B50] Qiu F. Zheng Y. Zhang Z. Xu S. (2007). Mapping of QTL associated with waterlogging tolerance during the seedling stage in maize. Ann. Bot. 99, 1067–1081. doi: 10.1093/aob/mcm055 17470902 PMC3244372

[B51] Rocha M. Licausi F. Araújo W. L. Nunes-Nesi A. Sodek L. Fernie A. R.. (2010). Glycolysis and the tricarboxylic acid cycle are linked by alanine aminotransferase during hypoxia induced by waterlogging of lotus japonicus. Plant Physiol. 152, 1501–1513. doi: 10.1104/pp.109.150045 20089769 PMC2832266

[B52] Setter T. L. Waters I. (2003). Review of prospects for germplasm improvement for waterlogging tolerance in wheat, barley and oats. Plant Soil. 253, 1–34. doi: 10.1023/A:1024573305997

[B53] Steffens B. Geske T. Sauter M. (2011). Aerenchyma formation in the rice stem and its promotion by H_2_O_2_ . New Phytol. 190, 369–378. doi: 10.1111/j.1469-8137.2010.03496.x 21039565

[B54] Steffens B. Rasmussen A. (2016). The physiology of adventitious roots. Plant Physiol. 170, 603–617. doi: 10.1104/pp.15.01360 26697895 PMC4734560

[B55] Steffens B. Sauter M. (2005). Epidermal cell death in rice (*Oryza sativa* l.) is regulated by ethylene, gibberellin and abscisic acid. Plant Physiol. 139, 713–721. doi: 10.1104/pp.105.064469 16169967 PMC1255990

[B56] Thomson C. J. Armstrong W. Waters I. Greenway H. (1990). Aerenchyma formation and associated oxygen movement in seminal and nodal roots of wheat. Plant Cell Environ. 13, 395–403. doi: 10.1111/j.1365-3040.1990.tb02144.x

[B57] Thomson C. J. Colmer T. D. Watkins E. L. J. Greenway H. (1992). Tolerance of wheat (*Triticum aestivum* cvs. gamenya and kite) and triticale (*Triticosecale* cv. Muir) to waterlogging. New Phytol. 120, 335–344. doi: 10.1111/j.1469-8137.1992.tb01073.x

[B58] Tong C. Hill C. B. Zhou G. Zhang X. Q. Li C. (2021). Opportunities for improving waterlogging tolerance in cereal crops-physiological traits and genetic mechanisms. Plants. 10, 1560. doi: 10.3390/plants10081560 34451605 PMC8401455

[B59] Toojinda T. Siangliw M. Tragoonrung S. Vanavichit A. (2003). Molecular genetics of submergence tolerance in rice: QTL analysis of key traits. Ann. Bot. 91, 243–253. doi: 10.1093/aob/mcf072 12509344 PMC4244984

[B60] Trought M. C. T. Drew M. (1980). The development of waterlogging damage in wheat seedlings (*Triticum aestivum* l.). Plant Soil. 56, 187–199. doi: 10.0032-079X/80/0563-0187S01.95

[B61] Vidoz M. L. Loreti E. Mensuali A. Alpi A. Perata P. (2010). Hormonal interplay during adventitious root formation in flooded tomato plants. Plant J. 63, 551–562. doi: 10.1111/j.1365-313x.2010.04262.x 20497380

[B62] Visser E. J. W. Cohen J. D. Barendse G. W. M. Blom C. W. P. M. Voesenek L. A. C. J. (1996). An ethylene-mediated increase in sensitivity to auxin induces adventitious root formation in flooded rumex palustris Sm. Plant Physiol. 112, 1687–1692. doi: 10.1104/pp.112.4.1687 12226472 PMC158102

[B63] Voesenek L. Bailey-Serres J. (2013). Flooding tolerance: O_2_ sensing and survival strategies. Curr. Opin. Plant Biol. 16, 647–653. doi: 10.1016/j.pbi.2013.06.008 23830867

[B64] Wei X. Xu H. Rong W. Ye X. Zhang Z. (2019). Constitutive expression of a stabilized transcription factor group VII ethylene response factor enhances waterlogging tolerance in wheat without penalizing grain yield. Plant Cell Environ. 42, 1471–1485. doi: 10.1111/pce.13505 30566765

[B65] Wiengweera A. Greenway H. Thomson C. J. (1997). The use of agar nutrient solution to simulate lack of convection in waterlogged soils. Ann. Bot. 80, 115–123. doi: 10.1006/anbo.1996.0405

[B66] Xu L. Pan R. Shabala L. Shabala S. Zhang W. Y. (2019). Temperature influences waterlogging stress-induced damage in arabidopsis through the regulation of photosynthesis and hypoxia-related genes. Plant Growth Regul. 89, 143–152. doi: 10.1007/s10725-019-00518-x

[B67] Xu Q. T. Yang L. Zhou Z. Q. Mei F. Z. Qu L. H. Zhou G. S. (2013). Process of aerenchyma formation and reactive oxygen species induced by waterlogging in wheat seminal roots. Planta. 238, 969–982. doi: 10.1007/s00425-013-1947-4 23975011

[B68] Yamauchi T. Watanabe K. Fukazawa A. Mori H. Abe F. Kawaguchi K.. (2014). Ethylene and reactive oxygen species are involved in root aerenchyma formation and adaptation of wheat seedlings to oxygen-deficient conditions. J. Exp. Bot. 65, 261–273. doi: 10.1093/jxb/ert371 24253196 PMC3883296

[B69] Yin L. L. Zhang H. Tang Z. S. Xu J. Y. Yin D. Zhang Z. W.. (2021). rMVP: a memory-efficient, visualization-enhanced, and parallel-accelerated tool for genome-wide association study. Genom proteom bioinf. 19, 619–628. doi: 10.1016/j.gpb.2020.10.007 PMC9040015 33662620

[B70] Yu M. Chen G. Y. (2013). Conditional QTL mapping for waterlogging tolerance in two RILs populations of wheat. SpringerPlus. 2, 1–7. doi: 10.1186/2193-1801-2-245 23750334 PMC3671099

[B71] Yu M. Mao S. L. Chen G. Y. Liu Y. X. Li W. Wei Y. M.. (2014). QTLs for waterlogging tolerance at germination and seedling stages in population of recombinant inbred lines derived from a cross between synthetic and cultivated wheat genotypes. J. Integr. Agric. 13, 31–39. doi: 10.1016/S2095-3119(13)60354-8

[B72] Zeng F. Konnerup D. Shabala L. Zhou M. Colmer T.D. Zhang G.. (2014). Linking oxygen availability with membrane potential maintenance and K+ retention of barley roots: implications for waterlogging stress tolerance. Plant Cell Environ. 37, 2325–2338. doi: 10.1111/pce.12422 25132404

[B73] Zhang X. Shabala S. Koutoulis A. Shabala L. Johnson P. Hayes D.. (2015). Waterlogging tolerance in barley is associated with faster aerenchyma formation in adventitious roots. Plant Soil. 394, 355–372. doi: 10.1007/s11104-015-2536-z

[B74] Zhang X. Zhou G. Shabala S. Koutoulis A. Shabala L. Johnson P.. (2016). Identification of aerenchyma formation-related QTL in barley that can be effective in breeding for waterlogging tolerance. Theor. Appl. Genet. 129, 1167–1177. doi: 10.1007/s00122-016-2693-3 26908252

[B75] Zhou M. (2011). Accurate phenotyping reveals better QTL for waterlogging tolerance in barley. Plant Breed. 130, 203–208. doi: 10.1111/j.1439-0523.2010.01792.x

[B76] Zhu C. S. Gore M. Buckler E. S. Yu J. M. (2008). Status and prospects of association mapping in plants. Plant Genome. 1, 5–20. doi: 10.3835/plantgenome2008.02.0089

