# Supplementary figures and images for: Genome-wide association study reveals quantitative trait loci for waterlogging-triggered adventitious roots and aerenchyma formation in common wheat
Source: Front Plant Sci. 2022 Nov 23;13:1066752. doi: 10.3389/fpls.2022.1066752 (PMC9727299; doi:10.3389/fpls.2022.1066752)

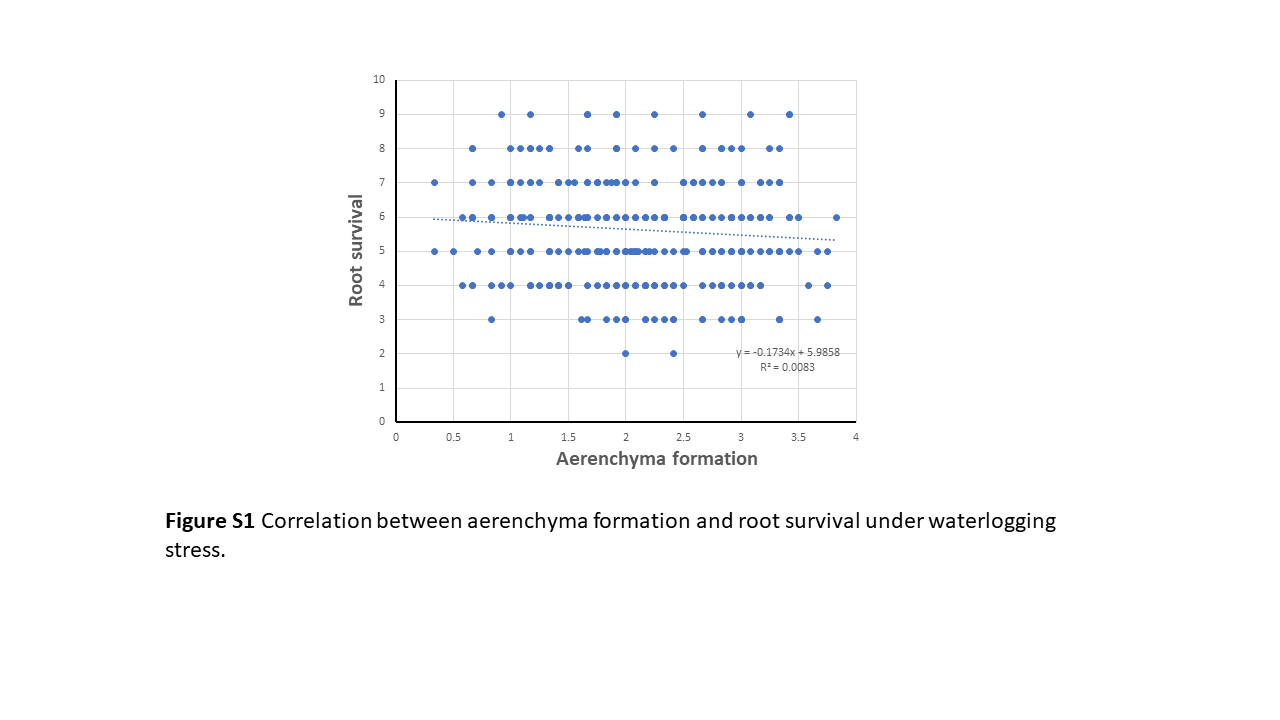

Supplement: Supplementary file 2 [file Image_1.tif]

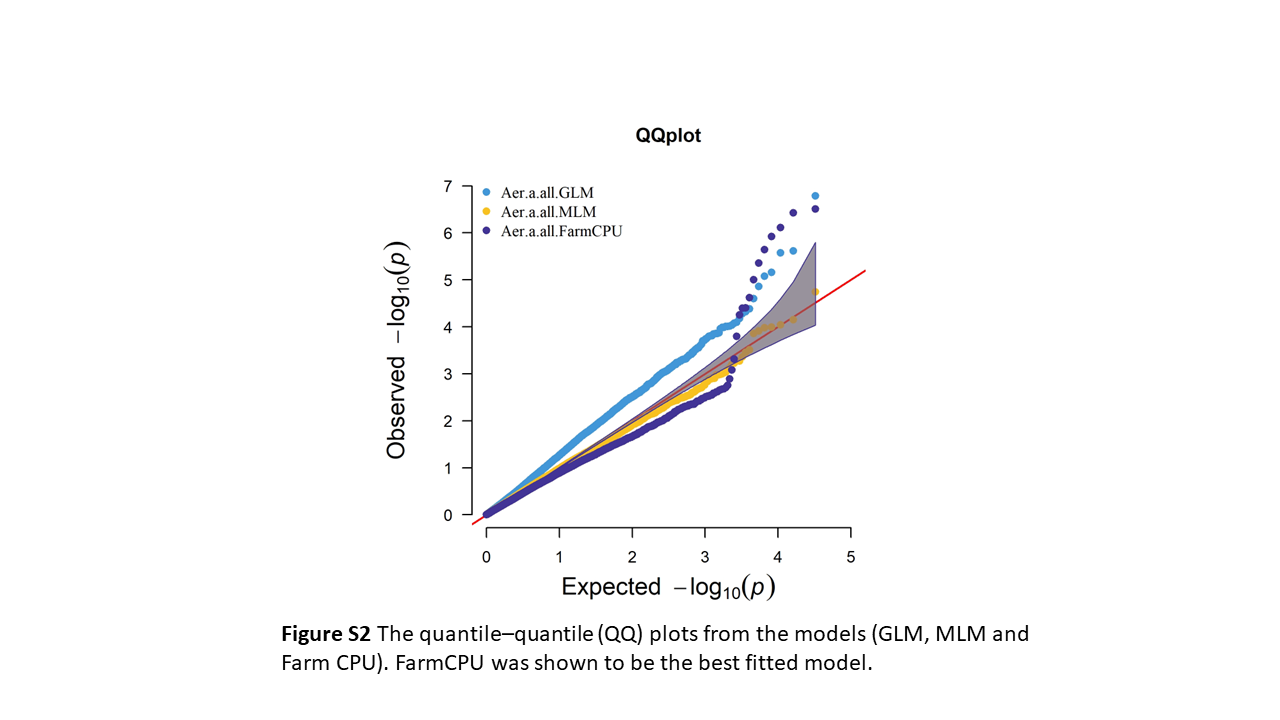

Supplement: Supplementary file 3 [file Image_2.tif]
